# Renin‐Angiotensin System Inhibitor Use and Mortality in Older Hypertensive Patients With and Without Dementia: A Nationwide Registry‐Based Cohort Study

**DOI:** 10.1111/jch.70350

**Published:** 2026-08-21

**Authors:** Marta Villa Lopez, Minh Tuan Hoang, Hong Xu, Jakob Norgren, Bojana Petek, Juraj Secnik, Irena Kalar, Dorota Religa, Silvia Maioli, Minjia Mo, Ingemar Kåreholt, Kristina Johnell, Maria‐Luz Cuadrado, Maria Eriksdotter, Sara Garcia‐Ptacek

**Affiliations:** ^1^ Division of Clinical Geriatrics, Department of Neurobiology, Care Sciences and Society Karolinska Institutet Stockholm Sweden; ^2^ Department of Medicine, School of Medicine Universidad Complutense Madrid Spain; ^3^ Division of Neurology, Department of Medicine University of Alberta Hospital Edmonton Alberta Canada; ^4^ Department of Medical Epidemiology and Biostatistics Karolinska Institutet Stockholm Sweden; ^5^ Division of Neurogeriatrics, Department of Neurobiology, Care Sciences and Society, Karolinska Institutet Stockholm Sweden; ^6^ Clinical Institute of Genomic Medicine University Medical Centre Ljubljana Ljubljana Slovenia; ^7^ Faculty of Medicine University of Ljubljana Ljubljana Slovenia; ^8^ Department of Neurology, Charles University, Second Faculty of Medicine Motol University Hospital Prague Czech Republic; ^9^ Department of Neurology University Medical Centre Ljubljana Ljubljana Slovenia; ^10^ Theme Inflammation and Aging Karolinska University Hospital Stockholm Sweden; ^11^ Aging Research Center Karolinska Institutet and Stockholm University Stockholm Sweden; ^12^ Institute of Gerontology, School of Health and Welfare Jönköping University Jönköping Sweden; ^13^ Department of Neurology Hospital Clínico San Carlos Madrid Spain

**Keywords:** angiotensin converting enzyme inhibitors, angiotensin receptor blockers, dementia, mortality, renin‐angiotensin system inhibitors

## Abstract

Cardiovascular risk factors are often undertreated in patients with dementia. Renin‐angiotensin system inhibitors (RASI) are first‐line treatments for hypertension. We examined the association between RASI and all‐cause mortality in hypertensive patients with and without dementia, comparing angiotensin‐converting enzyme inhibitors (ACEI) to angiotensin receptor blockers (ARB); and selected individual agents.

This nationwide register‐based cohort study included data from Swedish national registries 2007–2018. The study included 158 176 hypertensive patients with exposure to RASI medications (21 152 RASI users and 137,024 non‐users), after excluding prevalent users.

Propensity score matching was used to balance measured confounders. Cox regressions compared mortality risk among RASI users versus non‐users, ACEI users versus ARB users, and users of enalapril, ramipril, losartan, and candesartan versus other RASI users.

RASI use was associated with lower all‐cause mortality (hazard ratio[HR] 0.93, 95% confidence interval[CI] 0.91–0.95, *p*< 0.001) in the full cohort, and among patients with dementia (HR 0.88, 95% CI 0.85–0.92). Compared to ACEI, ARB were associated with lower mortality in the full cohort (HR 0.90, 95% CI 0.85–0.96), but results were not significant in patients with dementia. The comparison of the individual drugs were consistent with class‐level findings.

RASI use was associated with lower all‐cause mortality among older hypertensive patients, including those with dementia. These observational findings support continued attention to evidence‐based hypertension treatment in patients with dementia.

## 1 Introduction

Hypertension is a leading risk factor for all‐cause mortality worldwide, resulting in approximately 10.4 million deaths annually [1]. Renin‐angiotensin system inhibitors (RASI) are among the first‐line treatments for hypertension, and include angiotensin converting enzyme inhibitors (ACEI) and angiotensin receptor blockers (ARB) [2, 3].

Dementias present a high burden of morbimortality worldwide [4], but are often underestimated as causes of death [5]. The prevalence of both dementia and hypertension are expected to increase over the next few decades owing to population ageing [6, 7]. Dementia prevalence is projected to nearly triple, from 57 million affected individuals in 2019 to 153 million in 2050 [7]. These conditions pose significant public health concerns, particularly given their association. There is strong epidemiological evidence that hypertension contributes to the development of dementia, including vascular and Alzheimer's dementia, and that treating hypertension can reduce dementia risk [8, 9]. Less evidence exists on how antihypertensive treatment can affect dementia progression and mortality.

Previous studies comparing different antihypertensives have often found no significant differences in mortality [10, 11]. ACEI have rarely been compared head‐to‐head against ARB [12], but epidemiological studies suggest potential benefit of ACEI for mortality in some cohorts or subpopulations [13], while others find lower mortality with ARB [14, 15]. However, data is scarce in specific populations such as aged individuals or persons with dementia. To our knowledge, no prior studies examine mortality with RASI specifically in patients with dementia.

The objective of this study was to determine whether RASI presented with similar mortality associations in hypertensive patients with and without dementia. Second, we aimed to compare mortality in ACEI versus ARB. Finally, we examined the most frequently prescribed individual medications in this cohort, consisting of enalapril, ramipril, losartan and candesartan and compared them to other RASI medications.

## 2 Methods

### 2.1 Data Sources

We conducted a nationwide cohort study in Sweden, selecting hypertensive patients with dementia and non‐dementia controls from quality and compulsory national registries merged with the Swedish personal identification number, between 2007 and 2018. Data sources included the national quality registry SveDem (Swedish Registry for Cognitive/Dementia Disease), launched in 2007, capturing a substantial proportion (28%–43%) of the presumed dementia incidence (defined as including both diagnosed and undetected dementia cases) [16]. The Swedish National Patient Register (NPR) records specialist care and hospitalization diagnoses coded using ICD‐10 (International Statistical Classification of Diseases and Related Health Problems). The Prescribed Drug Register (PDR) includes all medications dispensed in Swedish pharmacies with a total coverage close to 100% [17, 18]. The Swedish Total Population Register provides information on mortality and includes 100% of the Swedish population [19]. The Swedish Longitudinal Integrated Database for Health Insurance and Labor Market Studies (LISA) provided socioeconomic data, extracted in the year prior to dementia diagnosis [20]. Mortality data were collected until December 31st, 2018.

### 2.2 Cohort Definition

**FIGURE 1:**
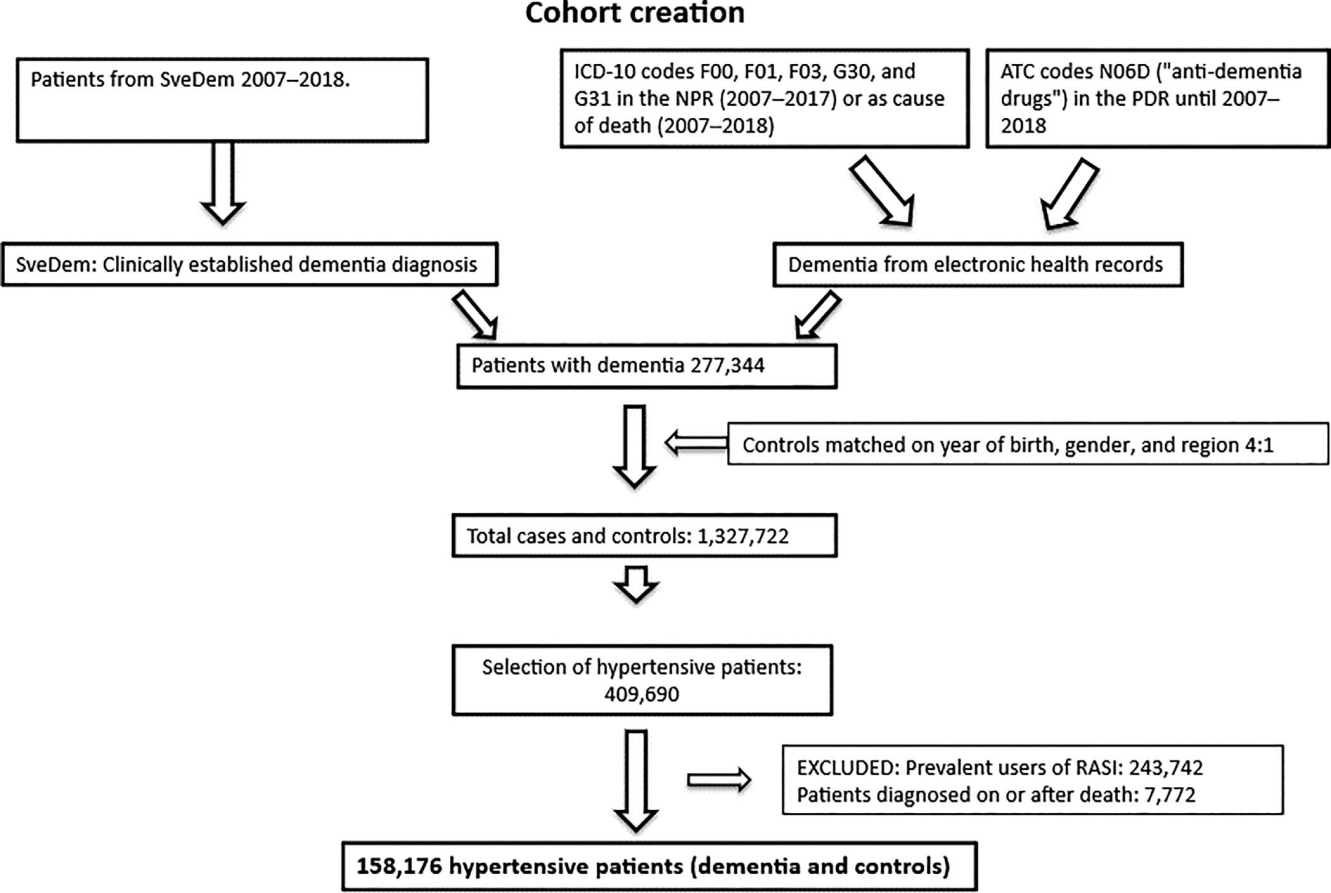
Patient selection flowchart. Patients with dementia were identified through SveDem (clinically confirmed dementia), through diagnoses or as a cause of death, and through electronic health records on prescriptions, diagnoses and causes of death (group of suspected dementia cases). This group was matched to up to four controls. Patients without hypertension were removed. Lastly, prevalent users of renin‐angotensin system inhibitors (RASI) and those whose dementia diagnosis was on or after the date of death were excluded. Prevalent users were defined as those who had a prescription earlier than one year before inclusion date. ACEIs, angiotensin converting enzyme inhibitors; ARBs, angiotensin II receptor blockers; ATC, anatomical therapeutic chemical code; ICD: International Classification of Diseases; NPR, National Patient Register; PDR, Prescribed Drug Register; SveDem, Swedish registry for cognitive disorders and dementia.

From SveDem, we identified a total of 79 711 individuals with dementia. To improve case ascertainment, additional dementia cases were identified through the NPR and Cause of Death Registry (ICD‐10 codes F00, F01, F03, G30 and G31), and dementia‐specific medications (cholinesterase inhibitors and memantine) from the PDR (ATC codes N06D). This added 197 633 patients with dementia ascertained through electronic health records (EHR). The Swedish Board of Health and Welfare then extracted up to four controls, matched by age (± 3 years), sex and region, for individuals with dementia identified in either SveDem or through electronic health records, for a total base cohort of 1 327 722 individuals (Figure 1).

From this cohort, we selected individuals with hypertension, defined by ICD‐10 codes I10‐15, H35.0, and I67.4, resulting in 409 690 individuals for analyses.

### 2.3 Exposure Definition

The main exposures were incident use of RASI, ACEI and ARB during the year preceding the index date, defined through ATC codes (Supplementary eTable 1). The index date was the date of dementia diagnosis for cases and the corresponding matched date for controls. Incident use was defined as at least one dispensation of RASI during the 12 months before the index date, with no recorded RASI dispensation between the preceding 12‐month washout period. Users of RASI during the 12–24 months preceding index date were considered prevalent users (*n* = 243 742), and were excluded (Figure 1). Exposures were defined at cohort entry at baseline and fixed throughout follow‐up. Baseline date was the date of cases diagnosed with dementia, and for controls, the corresponding date for their matching dementia case. The most frequently prescribed RASI (enalapril, ramipril, candesartan, losartan) were additionally compared to other RASI. Dispensed medications were obtained from the PDR with ATC codes shown in Supplementary eTable 1.

### 2.4 Confounding Control

Analyses were controlled for age, sex, and income and education which were extracted from LISA. Comorbidities and Charlson Comorbidity Index were obtained from the NPR (Supplementary eTable 2). Co‐medications (from the PDR) were also used for confounding control (Supplementary eTable 3). Hypertension with target organ damage (ICD‐10 diagnosis) was extracted separately for control, as blood pressure measurements were not available. Covariates were ascertained before or at the index date, and not updated during follow‐up.

### 2.5 Outcomes

All‐cause mortality was ascertained through the Total Population Registry. Patients whose dementia diagnosis date coincided with or occurred after their date of death (*n* = 7772) were excluded.

### 2.6 Statistical Analysis

**FIGURE 2:**
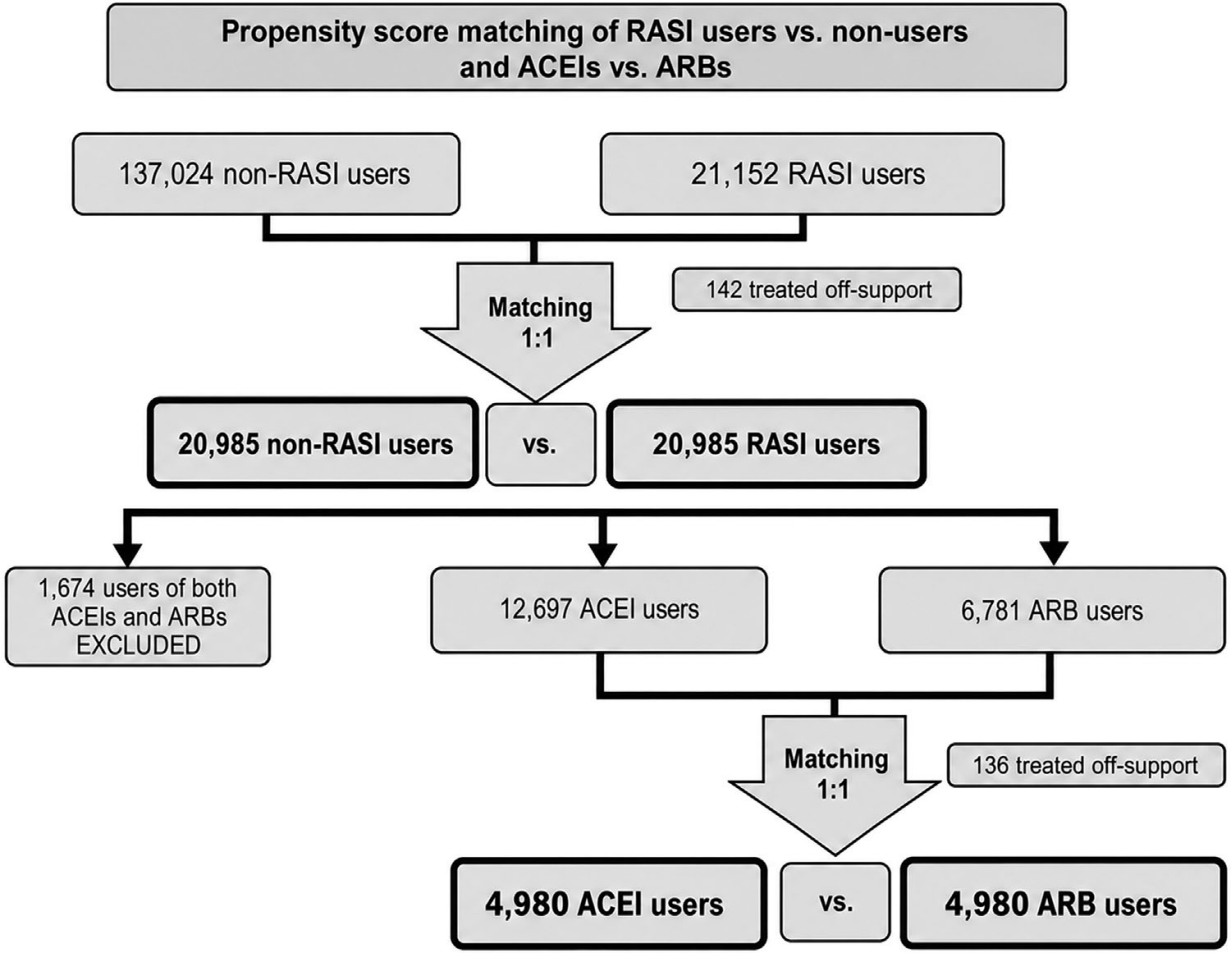
Propensity score matching exposed and unexposed cohorts. The total 158 176 patients who met the inclusion criteria (hypertension diagnosis, non‐prevalent‐RASI users), were divided into RASI and non‐RASI users. This whole cohort was matched 1:1 using propensity score with no replacement, leaving 20985 non‐RASI users who were compared to 20985 RASI users. In a separate step, ARB users were matched with ACEI users, leaving 4980 patients in each group. ACEIs, angiotensin converting enzyme; RASI, renin angiotensin system inhibitors.

Propensity‐score matching was performed using 1:1 nearest‐neighbor matching without replacement, a caliper of 0.001, and restriction to common support (Figure 2). Separate propensity scores were estimated for each exposure: (1) RASI users versus non‐users, (2) ARB versus ACEI users and (3) Between individual medication users versus other RASI users. Separate scores were estimated for the subgroup analyses of dementia patients. Propensity scores were estimated with logistic regression models predicting treatment assignment. Candidate variables were selected based on clinical relevance, prior literature on association with treatment use, and literature and our previous experience with mortality predictors in SveDem [21, 22, 23]. The distributions of the propensity of treatment between cases and controls were checked graphically. Once patients were matched, we checked the standardized difference (SD) between the groups to confirm these differences were under 0.1. The final propensity scores included age, sex, dementia category (none, SveDem, dementia from EHR), socioeconomics, Charlson Comorbidity Index, comorbidities and comedications (Table 1).

**TABLE 1:** Descriptive characteristics of the whole cohort (i.e., dementia + controls) and the propensity‐score matched cohort (RASI exposed vs. non‐exposed).

	Total cohort (*n* = 158 176)			Matched cohort (*n* = 41970)		
	**Non‐users** **(*n* = 137 024)**	**RASI** **(*n* = 21 152)**	**SD**	**Non‐users** **(*n* = 20985)**	**RASI** **(*n* = 20985)**	**SD**
**Age, median (IQR)**	84.1 (79.1, 88.7)	82.2 (77.2, 86.7)	−0.271	82.3 (77.0, 87.0)	82.3 (77.2, 86.7)	0.002
**Women, *n* (%)**	86 557 (63.2)	12 281 (58.1)	−0.105	12214 (58.2%)	12222 (58.2%)	0.001
**Dementia**						
No dementia	94 631 (69.1)	14 970 (70.8)	0.038	14793 (70.5%)	14856 (70.8%)	0.007
SveDem	30 863 (22.5)	4162 (19.7)	−0.070	4166 (19.9%)	4126 (19.7%)	−0.005
EHR dementia	11 530 (8.4)	2020 (9.5)	0.040	2026 (9.7%)	2003 (9.5%)	−0.004
**Diagnosis year, median (IQR)**	2013 (2010, 2015)	2012 (2010, 2015)	−0.062	2012.0 (2010.0, 2015.0)	2012.0 (2010.0, 2015.0)	0.008
**Income, median (IQR)**	1477.4 (1246.5, 1769.7)	1489.2 (1249.6, 1809.5)	0.006	1494.4 (1256.4, 1811.6)	1488.9 (1249.7, 1809.3)	−0.002
**Education**						
≤9 years	73 595 (53.7)	10 947 (51.8)	−0.038	10902 (52.0%)	10873 (51.8%)	−0.003
High school	50 436 (36.8)	8041 (38)	0.026	7920 (37.7%)	7988 (38.1%)	0.007
University	9857 (7.2)	1646 (7.8)	0.023	1677 (8.0%)	1636 (7.8%)	−0.007
Missing	3136 (2.3)	518 (2.4)	0.006	486 (2.3%)	488 (2.3%)	0.001
**Marital status, *n* (%)**						
Divorced/ widowed	76 632 (55.9)	10 926 (51.7)	−0.085	10951 (52.2%)	10872 (51.8%)	−0.008
Married	50 722 (37)	8662 (41)	0.081	8475 (40.4%)	8591 (40.9%)	0.011
Single	9614 (7)	1540 (7.3)	0.011	1559 (7.4%)	1522 (7.3%)	−0.007
**Place of birth, *n* (%)**						
Sweden or other Nordics	129 813 (94.8)	19 739 (93.4)	−0.057	19583 (93.3%)	19620 (93.5%)	0.007
Europe	5266 (3.8)	964 (4.6)	0.036	980 (4.7%)	948 (4.5%)	−0.007
Other	1887 (1.4)	424 (2)	0.049	422 (2.0%)	417 (2.0%)	−0.002
**Comorbidities, *n* (%)**						
Diabetes mellitus	22 909 (16.7)	4313 (20.4)	0.094	4248 (20.2%)	4284 (20.4%)	0.004
Heart failure	19 719 (14.4)	5517 (26.1)	0.294	5407 (25.8%)	5374 (25.6%)	−0.004
Myocardial infarction	13 889 (10.1)	3656 (17.3)	0.209	3584 (17.1%)	3532 (16.8%)	−0.007
Acute kidney injury	3065 (2.2)	532 (2.5)	0.018	519 (2.5%)	528 (2.5%)	0.003
Stroke	35 420 (25.8)	5790 (27.4)	0.035	5797 (27.6%)	5747 (27.4%)	−0.005
Atrial fibrillation	33 823 (24.7)	6468 (30.6)	0.132	6406 (30.5%)	6379 (30.4%)	−0.003
Alcohol‐related diagnoses	3240 (2.4)	529 (2.5)	0.009	522 (2.5%)	525 (2.5%)	0.001
Cancer	50 260 (36.7)	7233 (34.2)	−0.052	7293 (34.8%)	7187 (34.2%)	−0.011
Liver Disease	1885 (1.4)	256 (1.2)	−0.015	268 (1.3%)	254 (1.2%)	−0.006
Hypertension with organ damage	7627 (5.6)	1335 (6.3)	0.032	1347 (6.4%)	1325 (6.3%)	−0.004
Charlson Comorbidity Index, median (IQR)	2.0 (1.0, 3.0)	2.0 (1.0, 3.0)	0.081	2.0 (1.0, 3.0)	2.0 (1.0, 3.0)	−0.006
**Medications, *n* (%)**						
Cardiac drugs	4453 (3.2)	726 (3.4)	0.010	710 (3.4%)	721 (3.4%)	0.003
Diuretics	71 309 (52)	12 170 (57.5)	0.111	11935 (56.9%)	12036 (57.4%)	0.010
Beta blockers	77 949 (56.9)	13 680 (64.7)	0.160	13602 (64.8%)	13529 (64.5%)	−0.007
Calcium Channel Blockers	53 654 (39.2)	8841 (41.8)	0.054	8840 (42.1%)	8772 (41.8%)	−0.007
Statins	41 131 (30)	8752 (41.4)	0.239	8575 (40.9%)	8610 (41.0%)	0.003
Anticoagulants	19 281 (14.1)	4309 (20.4)	0.167	4188 (20.0%)	4217 (20.1%)	0.003
Antiplatelets	66 062 (48.2)	11 865 (56.1)	0.158	11676 (55.6%)	11724 (55.9%)	0.005
Insulin	8127 (5.9)	1632 (7.7)	0.070	1615 (7.7%)	1616 (7.7%)	0.000
Hypnotics	44 803 (32.7)	6777 (32)	−0.014	6803 (32.4%)	6722 (32.0%)	−0.008
Anxiolytics	28 307 (20.7)	4094 (19.4)	−0.033	4093 (19.5%)	4060 (19.3%)	−0.004
Antidepressants	35 708 (26.1)	4797 (22.7)	−0.079	4877 (23.2%)	4774 (22.7%)	−0.012
Acetylcholinesterase inhibitors	3095 (2.3)	570 (2.7)	0.028	567 (2.7%)	566 (2.7%)	−0.000
Memantine	693 (0.5)	94 (0.4)	−0.009	113 (0.5%)	93 (0.4%)	−0.014

*Note*: The propensity score was calculated using the following variables: age, sex, dementia category (none, SveDem, electronic health records diagnosis), diagnosis year, income, education, marital status, region of birth, Charlson Comorbidity Index, high blood pressure with target organ damage, diabetes, heart failure, acute kidney injury, myocardial infarction, stroke, atrial fibrillation, alcohol‐related conditions, cancer, liver disease, antiplatelets, anticoagulants, cardiovascular drugs, beta‐blockers, diuretics, calcium channel blockers, statins, cholinesterase inhibitors, memantine, insulin, hypnotics, anxiolytics, and clinically relevant interactions between covariates.

Abbreviations: EHR dementia, dementia suspected through electronic health records including in‐hospital and specialist diagnosis, prescriptions of antidementia medications and death certificates; IQR, interquartile range; RASI, renin‐angiotensin system inhibitors, SD, standardized differences, SveDem, patients registered in the Swedish Registry for Cognitive/Dementia Disorders.

Dementia from electronic health records: patients identified through prescriptions, in‐hospital or specialist diagnoses and causes of death.

Survival analyses were performed in the matched cohorts using Cox proportional‐hazards models (STATA *stcox*). The Schoenfeld residual test and visual log‐minus‐log survival curves were used to determine whether hazards were proportional. Analyses were conducted separately across dementia strata. Sensitivity analyses with stratification by age, sex and diagnosis year were performed.

Analyses were performed using the Statistical Package for the Social Sciences software v.26 (IBM Corporation, Armonk, NY, USA) and SAS v. 9.4 (SAS Institute, Cary, NC, USA) and STATA version 14.2 (StataCorp, College Station, TX).

### 2.7 Ethics

Our study complies with the latest version of the Declaration of Helsinki and was approved by the Regional Ethical Review Board of Stockholm, Sweden (dnr 2017/501‐31). Before inclusion in SveDem, patients and/or caregivers were informed and could decline or withdraw participation. According to Swedish legislation, NPR, PDR, and the Total Population Registry are mandatory.

## 3 Results

After removing prevalent RASI users and patients whose dementia diagnosis date coincided with or occurred after death, a total of 158 176 patients remained for analyses (Figure 1). As shown in Table 1, RASI users were younger, more often male and had a higher prevalence of heart failure (26% vs. 14% standardized difference—SD—0.294), history of myocardial infarction (17% vs. 10%, SD 0.209) and atrial fibrillation (31 vs. 25%, SD 0.132). Figure 2 shows the process of propensity score matching: restriction to common support excluded 142 RASI users (0.7%) and no non‐users. The final matched cohort included 41 970 individuals: 20 985 RASI users and 20 985 non‐users. Post‐matching, the exposed and unexposed groups showed no relevant differences in variables affecting mortality (Table 1).

Table 2 shows the matched cohort of ARB versus ACEI. For this comparison, individuals who took both ACEI and ARBs within the exposure period were excluded (*n* = 1674). Propensity score matching yielded 4980 patients in each arm. The comparisons of the matched cohorts of enalapril, ramipril, losartan, and candesartan versus other RASI are shown in Supplementary eTables 4 to 7.

**TABLE 2:** Descriptive characteristics of the matched cohort of ARB users versus ACEI users.

	MATCHED COHORT (*n* = 9960)		
	**ACEI users** (*n* = 4980)	**ARB users** (*n* = 4980)	**SD**
**Age, median (IQR)**	81.9 (76.7, 86.4)	81.6 (76.7, 86.1)	0.011
**Women, *n* (%)**	2931 (58.9%)	2991 (60.1%)	0.025
**Dementia**			
No dementia	3703 (74.4%)	3707 (74.4%)	0.002
SveDem	833 (16.7%)	825 (16.6%)	−0.004
EHR dementia	444 (8.9%)	448 (9.0%)	0.003
**Diagnosis year, median (IQR)**	2014.0 (2011.0, 2016.0)	2014.0 (2011.0, 2016.0)	0.008
**Income, median (IQR)**	1516.8 (1260.6, 1846.3)	1524.4 (1274.7, 1853.0)	0.010
**Education, *n* (%)**			
≤9 years	2396 (48.1%)	2395 (48.1%)	−0.000
High school	2014 (40.4%)	2023 (40.6%)	0.004
University	452 (9.1%)	454 (9.1%)	0.001
Missing	118 (2.4%)	108 (2.2%)	−0.013
**Marital status, *n* (%)**			
Divorced/widowed	2470 (49.6%)	2489 (50.0%)	0.008
Married	2149 (43.2%)	2137 (42.9%)	−0.005
Single	361 (7.2%)	354 (7.1%)	−0.005
**Place of birth, *n* (%)**			
Sweden or other Nordics	4641 (93.2%)	4650 (93.4%)	0.007
Europe	206 (4.1%)	213 (4.3%)	0.007
Other countries	133 (2.7%)	117 (2.3%)	−0.021
**Comorbidities, *n* (%)**			
Diabetes mellitus	1039 (20.9%)	1040 (20.9%)	0.000
Heart failure	908 (18.2%)	921 (18.5%)	0.007
Myocardial infarction	558 (11.2%)	588 (11.8%)	0.019
Acute kidney injury	138 (2.8%)	132 (2.7%)	−0.007
Stroke	1307 (26.2%)	1282 (25.7%)	−0.011
Atrial fibrillation	1339 (26.9%)	1333 (26.8%)	−0.003
Alcohol‐related diagnoses	98 (2.0%)	112 (2.2%)	0.020
Cancer	1785 (35.8%)	1816 (36.5%)	0.013
Liver disease	68 (1.4%)	67 (1.3%)	−0.002
Hypertension with organ damage	338 (6.8%)	324 (6.5%)	−0.011
Charlson Comorbidity Index, median (IQR)	2.0 (1.0, 3.0)	2.0 (1.0, 3.0)	0.007
**Medications, *n* (%)**			
Cardiac drugs	173 (3.5%)	169 (3.4%)	−0.004
Diuretics	2701 (54.2%)	2695 (54.1%)	−0.002
Beta blockers	2974 (59.7%)	3032 (60.9%)	0.024
Calcium channel blockers	2258 (45.3%)	2262 (45.4%)	0.002
Statins	1998 (40.1%)	1965 (39.5%)	−0.014
Anticoagulants	961 (19.3%)	979 (19.7%)	0.009
Antiplatelets	2472 (49.6%)	2479 (49.8%)	0.003
Insulin	405 (8.1%)	399 (8.0%)	−0.004
Hypnotics	1558 (31.3%)	1600 (32.1%)	0.018
Anxiolytics	934 (18.8%)	937 (18.8%)	0.002
Antidepressants	1078 (21.6%)	1105 (22.2%)	0.013
Acetylcholinesterase inhibitors.	144 (2.9%)	144 (2.9%)	−0.000
Memantine	20 (0.4%)	26 (0.5%)	0.018

*Note*: The propensity score was calculated using the following variables: age, sex, dementia category (none, SveDem, electronic health records diagnosis), diagnosis year, income, education, marital status, region of birth, Charlson Comorbidity Index, high blood pressure with target organ damage, diabetes, heart failure, acute kidney injury, myocardial infarction, stroke, atrial fibrillation, alcohol‐related conditions, cancer, liver disease, antiplatelets, anticoagulants, cardiovascular drugs, beta‐blockers, diuretics, calcium channel blockers, statins, cholinesterase inhibitors, memantine, insulin, hypnotics, anxiolytics, and clinically relevant interactions between covariates.

Abbreviations: ACEIs, angiotensin‐converting enzyme inhibitors, ARBs, angiotensin‐II receptor blockers, EHR dementia: dementia suspected through electronic health records including in‐hospital and specialist diagnosis, prescriptions of antidementia medications and death certificates; IQR, Interquartile range; SD, standardized differences; SveDem: patients registered in the Swedish Registry for Cognitive/Dementia Disorders.

### 3.1 Risk of All‐Cause Mortality

**FIGURE 3:**
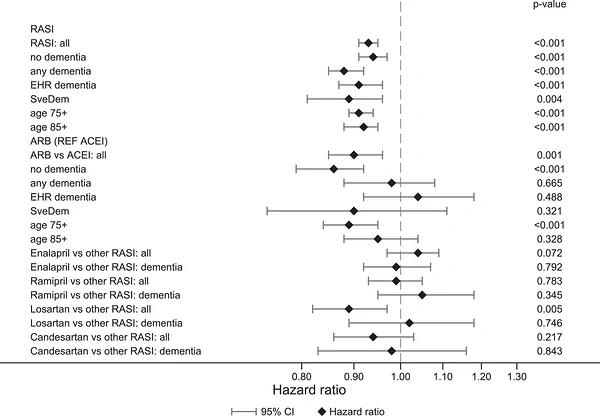
RASI use and all‐cause mortality. Hazard ratios (HR) and 95% confidence intervals (95% CIs) are presented. The propensity score was calculated using the following variables: age, sex, dementia category (none, SveDem, electronic health records diagnosis), diagnosis year, income, education, marital status, region of birth, Charlson Comorbidity Index, high blood pressure with target organ damage, diabetes, heart failure, acute kidney injury, myocardial infarction, stroke, atrial fibrillation, alcohol‐related conditions, cancer, liver disease, and medications. ACEI, angiotensin converting enzyme inhibitors; ARB, angiotensin receptor blockers; EHR dementia, Dementia from electronic health records: patients identified through prescriptions, in‐hospital or specialist diagnoses and causes of death; RASI, renin‐angiotensin system inhibitors. SveDem, patients registered in the Swedish Registry for Cognitive/Dementia Disorders.

During the follow‐up period of 3.4 years (standard deviation 2.7), 11555 (55.1%) of the RASI‐exposed and 12015 (57.3%) of the non‐exposed patients died. The formal Shoenfeld residual test was statistically significant in the matched cohort, but residual correlations were small and visual inspection of the log‐minus‐log survival curves didn't indicate major non‐proportionality; therefore, Cox proportional‐hazards models were retained. Mortality risk was significantly lower in the group exposed to RASI (HR = 0.93, 95% CI 0.91–0.95, Figure 3). This association was maintained in stratified analyses by dementia status: in patients without dementia RASI exposure was associated with HR 0.94 (95% CI 0.91–0.97) while in patients with dementia this HR was 0.88 (95% CI 0.85–0.92). Further stratifying by origin of dementia diagnosis (SveDem vs. EHR) yielded similar results.

In a second propensity score matched cohort, 4980 ACEI users were compared to 4980 ARB users. Among ACEI users in the matched cohort, 2278 (45.7%) died compared to 2119 (42.6%) ARB users. ARB users had lower mortality risk than ACEI users (HR = 0.90, 95% CI 0.85–0.96) (Figure 3). When stratifying by dementia status, this association remained in patients without dementia (HR 0.86, 95% CI 0.79–0.92) but was attenuated and became non‐significant in patients with dementia (HR 0.98, 95% CI 0.88–1.08). Stratifying by cohort age yielded similar results.

The most frequently used RASI medications in this cohort were enalapril, ramipril, losartan and candesartan. The associations of the individual drugs trended in the same direction as those found for the comparisons by drug classes (ACEI vs. ARB‐figure 3) but were not statistically significant with the exception of losartan in the full sample (HR 0.89, 95% CI 0.82–0.97). Non‐significant results were obtained when analyzing exclusively patients with dementia.

## 4 Discussion

### 4.1 Mortality in Patients With Hypertension

This observational study found that RASI exposure was associated with lower all‐cause mortality risk in hypertensive patients with and without dementia. The average age of the cohort was high (ca 82 years), since it was based on a dementia population with matched controls. Furthermore, results persisted in stratified analyses among the oldest patients in the cohort (75 years and above and 85 years and above).

Two Cochrane reviews have compared the morbidity and mortality of different antihypertensives [10, 11]. No significant differences were found in mortality from any cause comparing among different classes of antihypertensive medications. However, neither study directly compared ACEI to ARB. The randomized clinical trial ONTARGET found telmisartan to be equivalent to ramipril for a composite of death and cardiovascular events [12]. In type 2 diabetes, contradictory results are reported [14, 24]. A meta‐analysis of cardiovascular morbimortality including 20 clinical trials with a high proportion of hypertensive participants and compared different antihypertensive treatments. RASI treatment was associated with 5% reduction in all‐cause mortality, but this was driven by ACEI, while ARB showed no mortality benefit [13].

In our cohort of aged individuals with hypertension, ARB were associated with lower mortality risk than ACEI. Compared to previous studies, the longer follow‐up in our cohort, large patient group and high number of observed events (deaths) may have revealed small differences in mortality between the drug classes [11]. The analyses of enalapril, ramipril, losartan and candesartan, which were the individual drugs most prescribed in this cohort, were often non‐significant but trended in line with the class association. The comparison of ARB versus ACEI was not significant in the subgroup of patients with dementia: this may be due to a smaller sample size, differences in prescription or comorbidities, or the shorter expected survival among this population leaving less time to demonstrate potential differences.

ARB typically have fewer adverse effects, potentially leading to better adherence among patients. This would be particularly apparent in a cohort study such as ours which typically does not reach the same compliance levels as clinical trials. Our findings could represent differences between real world patients and the selected populations of clinical trials. However, we cannot exclude that unmeasured confounding is responsible for part or all of this discrepancy.

In dementia patients, several factors influence mortality risk, including age, cognition, sex, frailty, diabetes, previous cardiovascular events, education, type of dementia (non‐Alzheimer dementia correlates with higher mortality), or body mass index (BMI) [21, 22, 25, 26, 27, 28, 29, 30]. Our analysis included these variables, except BMI, due to missing information in a large proportion of the cohort. The attenuation of the ARB versus ACEI association in the dementia subgroup should be interpreted cautiously and could reflect reduced statistical power, prescribing differences, comorbidities or a combination of these factors.

To date, no studies have specifically analyzed all‐cause mortality in dementia patients taking RASI. Our results warrant further research to explore optimal treatment strategies. ARB have been suggested as favorable from a cognitive health perspective [31, 32, 33] and our findings are compatible with at least comparable survival among ARB users relative to ACEI. However, given the observational design of this study, these results should not be used alone to prioritize ARB over ACEI in individual treatment decisions.

### 4.2 Limitations

Cohort entry was determined at the time of dementia diagnosis or matched index date for controls, rather than the exact date of RASI initiation which is an important limitation. Initial control matching was performed by the Swedish Board of Health and Welfare based on dementia diagnosis and dementia diagnosis date. This was done to ensure anonymity and provide a dataset to be used for several different studies, but for this reason the resulting cohort was not centered on RASI prescription or RASI prescription date: alignment on those factors was performed later through propensity score matching.

This study does not attempt a formal target‐trial emulation of RASI initiation versus non‐initiation. Although prevalent users were excluded and drug use defined before cohort entry, imperfect treatment initiation alignment, eligibility and start of follow‐up may have introduced biases due to selection, immortal time or residual confounding. Determining exposure by dispensation of medication does not guarantee adherence, and polypharmacy may be a confounding factor. Using registry data introduces the risk of underdiagnosis for certain conditions, and exact date of occurrence of comorbidities is uncertain. The NPR includes specialist and hospital diagnoses but not primary care. Since hypertension is usually managed in primary care, this will have biased the cohort towards more severe cases and generalizability to mild/primary care managed hypertension is uncertain. Although we attempted to control the severity of hypertension by including a variable for hypertension with target organ damage we lacked recorded blood pressure measurements. Because absolute blood pressure values were unavailable, we could not distinguish between uncontrolled, controlled or treatment‐resistant hypertension. Similarly, we did not have access to biomarkers or postmortem pathology data to refine the dementia diagnoses or functional status information, which could have enhanced our analysis. Since covariates were anchored to index date, rather than treatment initiation, and not updated, some time‐varying confounders influencing prescription may have been incompletely captured. Dementia ascertainment differed between SveDem and other registry captured patients. Diagnoses from the patient, medication and death certificates may have less precise onset dates than SveDem diagnoses. Individuals whose dementia date coincided or followed death were excluded, but misclassification of dementia timing remains possible.

Despite these limitations, our findings offer epidemiological evidence supporting RASI use in dementia patients with the same indication as in other older individuals.

## 5 Conclusion

Our findings suggest that RASI may be associated with lower risk of mortality in individuals with hypertension, including those with dementia. ARB presented lower mortality risk compared to ACEI in the whole cohort but results were not statistically significant among the subgroup of patients with dementia. Future research should aim to confirm these findings, determine the optimal blood pressure reduction, and identify which medications offer the most benefits. In the absence of clinical trials specifically including older populations or patients with dementia, our findings support the view that dementia alone should not preclude evidence‐based hypertension treatment, while causal effects of RASI initiation require further study. Given the links between the renin‐angiotensin system, cognition, and Alzheimer's pathology [32, 33], investigating the cognitive impacts of these drugs is crucial for comprehensive treatment strategies.

## Author Contributions

*Study concept and design*: Marta Villa Lopez, Minh Tuan Hoang, Hong Xu, Bojana Petek, Maria‐Luz Cuadrado, Maria Eriksdotter, Sara Garcia‐Ptacek. *Acquisition of data*: Dorota Religa, Maria Eriksdotter, Kristina Johnell, Sara Garcia‐Ptacek. *Analysis*: Marta Villa Lopez, Sara Garcia‐Ptacek. *Interpretation*: Marta Villa Lopez, Minh Tuan Hoang, Hong Xu, Jakob Norgren, Bojana Petek, Juraj Secnik, Ingemar Kåreholt, Dorota Religa, Silvia Maioli, Minjia M, Irena Kalar, Kristina Johnell, Maria‐Luz Cuadrado, Maria Eriksdotter, Sara Garcia‐Ptacek. *Preparation of manuscript*: Marta Villa Lopez, Minh Tuan Hoang, Sara Garcia‐Ptacek.

## Funding

This project is supported by the Swedish Research Council (Garcia‐Ptacek 2022–01425, Xu 2022–01428), StratNeuro start‐up grant (the Strategic Research Area Neuroscience‐Karolinska Institutet, Umeå University and KTH, Xu), Karolinska Institutet Central Career Grants (Garcia‐Ptacek), Emil and Wera Cornell Foundation (Garcia‐Ptacek), Johanniterorden I Sverige/Swedish Order of St. John, the Center for Innovative Medicine Foundation (Xu, CIMED, FoUI‐963369 & 1002840), Åke Wibergs stiftelse (Xu M24‐0221), the National Institute for Neurological Research (Programme EXCELES, ID Project No. LX22NPO5107)—Funded by the European Union—Next Generation EU (Secnik), Karolinska Institutet Research Foundation (Mo 2024–02322), Swedish Research Council for Health, Working Life and Welfare (Forte, Mo 2024–01295), Demensförbundet (Garcia‐Ptacek), Alzheimerfonden (Garcia‐Ptacek AF‐1011818, Maioli AF‐1011030), King Gustaf V and Queen Victoria's Foundation (Maioli), Margaretha af Ugglas’ Foundation (Maioli), the Region Stockholm and Karolinska Institutet collaboration grants ALF, the Emil and Wera Cornell foundation (Garcia‐Ptacek), Karolinska Institutets Strategic Research Area in Epidemiology and Biostatistics (SFOepi) and the New Innovative Roads Call—a private initiative from the Leif Lundblad family and others (Garcia‐Ptacek, Maioli).

## Conflicts of Interest

All authors report no conflicts of interest. The authors are grateful to SveDem (www.svedem.se) for providing data for this study. We thank all patients, caregivers, reporting units and coordinators in SveDem as well as SveDem steering committee. SveDem is supported financially by the Swedish Associations of Local Authorities and Regions.

## Data Availability

No data are available. The entities responsible for the original data and the Swedish law do not allow for sharing of the data from the Swedish national registers. Researchers may independently apply to SveDem and the Swedish Board of Health and Welfare to access registry data.
